# Incidence and risk factors for venous thromboembolism in patients with pretreated advanced pancreatic carcinoma

**DOI:** 10.18632/oncotarget.24721

**Published:** 2018-03-30

**Authors:** Shunsuke Kondo, Mitsuhito Sasaki, Hiroko Hosoi, Yasunari Sakamoto, Chigusa Morizane, Hideki Ueno, Takuji Okusaka

**Affiliations:** ^1^ Department of Hepatobiliary and Pancreatic Oncology, National Cancer Center Hospital, Tokyo, Japan; ^2^ Department of Experimental Therapeutics, National Cancer Center Hospital, Tokyo, Japan; ^3^ Department of Hepatobiliary and Pancreatic Oncology, National Cancer Center Hospital East, Chiba, Japan

**Keywords:** fibrin degradation product, incidence, pancreatic carcinoma, survival, venous thromboembolism

## Abstract

Patients with pancreatic carcinoma are at an increased risk of venous thromboembolism (VTE), which is a major cause of morbidity and mortality in various types of cancer. The aim of this study was to determine the incidence and clinical significance of VTE in patients with pancreatic carcinoma, and to identify biomarkers for the detection of VTE in these patients. The eligibility criteria were chemo-naïve patients with primary pancreatic carcinoma, an Eastern Cooperative Oncology Group performance status of 0–2, and adequate organ function. All patients were screened for VTE using compression ultrasonography and dynamic computed tomography. The primary endpoint was the incidence of VTE, which we hypothesized would be between 10.0–20.0% for symptomatic and asymptomatic patients combined. Associations between clinical presentation and VTE were evaluated. VTE-associated markers were also investigated for their role in predicting prognosis. In total, 103 patients met the eligibility criteria. The overall cumulative incidence rate of VTE in patients with previously untreated pancreatic carcinoma was 16.5%. VTE occurrence was strongly associated with elevated serum D-dimer, fibrin degradation product, thrombin/antithrombin III complex, and prothrombin fragment 1 + 2 levels. The median overall survival time of VTE-positive and VTE-negative patients was 427 and 515 days, respectively. Approximately one-sixth of patients with advanced pancreatic carcinoma experienced VTE, although most were asymptomatic. Measurement of serum D-dimer, fibrin degradation product, thrombin/antithrombin III complex, and prothrombin fragment 1 + 2 levels may be useful for the early detection of VTE in patients with advanced pancreatic carcinoma.

## INTRODUCTION

Pancreatic carcinoma is one of the most lethal cancers. It represents the fourth leading cause of cancer-related death in developed countries [1]. As pancreatic carcinoma has a high propensity for both local invasion and distant metastasis, surgical treatment is precluded for most patients who present with an advanced stage of the disease. Despite many treatment advances that have improved the outcomes of some pancreatic carcinoma patients, standard therapy has been found to have only a modest beneficial impact on advanced-stage patients [2], as reflected in their 5-year overall survival (OS) of <5.0% [1].

Venous thromboembolism (VTE) significantly increases the mortality rate of cancer patients and reduces their quality of life. The overall incidence of symptomatic VTE in ambulatory patients with multiple cancers is approximately 3.0%. However, the risk of VTE increases 6-fold in outpatients receiving chemotherapy and in those with advanced-stage disease [3]. Cancer types with the highest incidence of VTE include advanced malignancies of the brain, pancreas, lungs, ovaries, and stomach [4–8]. Not only is VTE considered an independent negative prognostic factor [9, 10], but the ensuing reduction in quality of life can delay cancer treatment, lead to more frequent and prolonged hospitalization, and result in higher treatment costs.

Recent studies have shown that elevated levels of D-dimer are a poor marker of survival in patients with various types of malignancies, including lung, pancreatic, colorectal, and breast cancers. A relationship between high plasma D-dimer levels and a poor prognosis has also been reported in gynecological cancers, including ovarian, cervical, and endometrial cancers [10–16]. However, systematic studies are required to confirm the significance of these findings.

The aim of this study was to determine the incidence and clinical significance of VTE in patients with pancreatic carcinoma, and to identify biomarkers for the detection of VTE in these patients.

## RESULTS

### Study population

In total, 103 patients with chemotherapy-naïve pancreatic carcinoma were identified and included in the analysis. None of the patients had first-degree relatives with VTE from medical interview. The demographic and clinical characteristics of the patients are summarized in Table 1. Seventeen patients (16.5%) presented with VTE. There were no significant differences in the baseline characteristics between patients with and without VTE. Three patients with VTE were symptomatic (2 with lower leg pain and 1 with leg edema) and 14 patients with VTE were asymptomatic. The clinical characteristics of patients with and without VTE were also similar (Table 1). Deep vein thrombosis was the most common form of VTE (*n* = 16 patients [94.1%]; 2 iliofemoral and 14 isolated distal deep vein thrombosis), followed by pulmonary embolism (*n* = 1 patient; 5.9%) (Table 2).

**Table 1:** Baseline characteristics

Characteristics	Patients			*P*-value
	All	VTE-positive	VTE-negative	
	(*n* = 103)	(*n* = 17)	(*n* = 86)	
Age (years), median (range)	65 (36–81)	67 (36–79)	64 (42–81)	0.53
Sex, *n* (%)				0.23
Male	56 (54.4)	7 (41.2)	49 (57.0)	
Female	47 (45.6)	10 (58.8)	37 (43.0)	
BMI, mean (range)	20.3	20.0	20.4	0.34
	(14.9–28.7)	(16.3–24.6)	(14.9–28.7)	
ECOG PS, *n* (%)				0.65
0	45 (43.7)	6 (35.3)	38 (44.2)	
1	57 (55.3)	11 (64.7)	47 (54.7)	
2	1 (1.0)	0 (0.0)	1 (1.1)	
Stage, *n* (%)				0.10
Locally advanced	37 (35.9)	3 (17.6)	34 (39.5)	
Metastatic	66 (64.1)	14 (82.4)	52 (60.5)	
Primary site, *n* (%)				0.17
Pancreatic head	46 (44.7)	5 (29.4)	41 (47.7)	
Pancreatic body	39 (37.9)	6 (35.3)	33 (38.4)	
Pancreatic tail	18 (17.4)	6 (35.3)	12 (13.9)	
Comorbidities and VTE risk factors non-related to cancer, *n* (%)				0.59
Hypertension	22 (21.4)	5 (29.4)	17 (19.8)	
Hyperlipidemia	6 (5.8)	0 (0.0)	6 (7.0)	
Diabetes	15 (14.6)	2 (11.8)	13 (15.1)	
Infection	0 (0.0)	0 (0.0)	0 (0.0)	
Total bed rest with bathroom privileges for >3 days, *n* (%)	0 (0.0)	0 (0.0)	0 (0.0)	–
CAD, *n* (%)	1 (1.0)	0 (0.0)	1 (1.1)	–
Brinkman index, mean	280	345	268	0.47
Smoking status, *n* (%)	0.42			
Non-smoker	59 (57.2)	8 (47.1)	51 (59.3)	
Smoker	22 (21.4)	3 (17.6)	19 (22.1)	
Ex-smoker	22 (21.4)	6 (35.3)	16 (18.6)	
CA19-9 level (U/mL), median (range)	854.0 (1.0–356,700)	1,516.0 (39.0–356,700.0)	686.5 (1.0–6,780.0)	0.22

Abbreviations: BMI, body mass index; CA19-9, carbohydrate antigen 19-9; CAD, coronary artery disease; PS, performance status.

**Table 2:** Localization and distribution of venous thromboembolism

Localization	Patients, *n* (%)
Total	17 (16.5)
Pulmonary embolism	3 (2.9)
Iliofemoral DVT	2 (1.9)
Isolated distal DVT	14 (13.6)
Upper limb DVT	0 (0.0)
Other (portal vein thromboembolism)	1 (1.0)

Abbreviations: DVT, deep vein thrombosis.

### Risk factors for VTE

The plasma levels of various factors are shown and compared in Table 3. Patients with VTE had significantly higher D-dimer, fibrin degradation product (FDP), thrombin/antithrombin III complex (TAT III), prothrombin fragment 1 + 2 (F1 + 2), interleukin (IL)-6, IL-8, and granulocyte macrophage colony-stimulating factor levels than those without VTE (Table 3).

**Table 3:** Biomarkers of venous thromboembolism

Variable	VTE-positive	VTE-negative	*P*-value
CRP (mg/dL)	0.42 (0.04–6.64)	0.21 (0.02–10.21)	0.13
D-dimer (ng/mL)	3.9 (0.6–23.6)	0.9 (0.1–8.8)	<0.0001
FDP (μg/mL)	10.6 (2.9–55.7)	6.4 (2.0–45.6)	0.004
Fibrinogen (mg/dL)	254 (238–589)	340 (202–589)	0.15
TAT III (ng/mL)	7.9 (2.6–60.0)	2.9 (1.3–31.5)	<0.0001
Total PAI-1 (ng/mL)	20.0 (10.0–54.0)	18.0 (4.0–64.0)	0.14
F1 + 2 (pmol/L)	264 (248–1,050)	211 (63–995)	<0.0001
Factor 2 (%)	86 (66–114)	87 (0–122)	0.72
Factor 7 (%)	82 (57–108)	82 (36–142)	0.24
Factor 8 (%)	117 (64–200)	111 (49–200)	0.95
Factor 10 (%)	84 (58–121)	84 (31–121)	0.64
VEGF (pg/mL)	34.0 (20.0–103.0)	24.5 (18.0–236.0)	0.15
IL-10 (pg/mL)	0.41 (0.18–1.46)	0.36 (0.13–4.24)	0.32
IL-1b (pg/mL)	0.05 (0.02–0.29)	0.06 (0.00–0.38)	0.72
IL-6 (pg/mL)	3.08 (0.65–9.71)	1.37 (0.19–13.40)	0.01
IL-8 (pg/mL)	35.20 (11.10–85.50)	20.00 (6.80–336.00)	0.008
GM-CSF (pg/mL)	0.20 (0.05–5.98)	0.10 (0.00–4.90)	0.01
TNF-β	0.30 (0.13–0.65)	0.30 (0.02–0.62)	0.92
CCL2	254.0 (164.0–639.0)	252.0 (144.0–531.0)	0.66
CCL22	1,110.0 (727.0–2,070.0)	970.0 (308.0–8,020.0)	0.12
CCL3	15.5 (6.1–30.6)	15.6 (4.7–42.5)	0.99
CCL4	135.0 (45.0–339.0)	119.0 (42.0–661.0)	0.56

Abbreviations: CCL, C-C motif ligand; CRP, C-reactive protein; GM-CSF, granulocyte macrophage colony-stimulating factor; IL, interleukin; PAI-1, plasminogen activator inhibitor-1; TNF-β, tumor necrosis factor-beta; VEGF, vascular endothelial cell growth factor.

**Figure 1:**
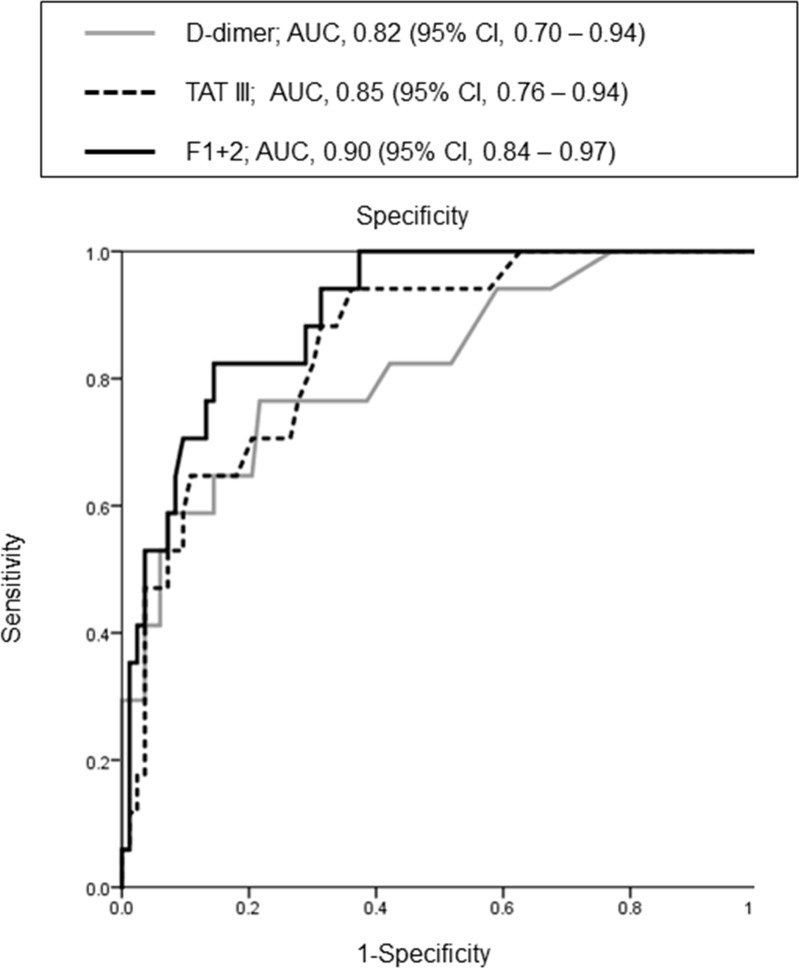
Receiver operating characteristic curve of the association between shown factors and venous thromboembolism

Receiver operating characteristic curve analysis was used to determine the accuracy of the estimations of VTE risk in patients with pancreatic carcinoma (Figure 1). The area under the curve for D-dimer was 0.82 (95.0% confidence interval [CI]: 0.70–0.94). The areas under the curve for TAT III (0.85 [95.0% CI: 0.76–0.94]) and F1 + 2 (0.90 [95.0% CI: 0.84–0.97]) also indicated that these factors were strongly associated with VTE risk.

### Survival analysis

The median OS of all patients was 504 (95.0% CI: 423–586) days. The median OS of patients with and without VTE was 427 (95.0% CI: 301–552) and 515 (95.0% CI: 424–607) days, respectively (*P* = 0.51). Elevated carbohydrate antigen 19-9 levels were associated with a poor prognosis (hazard ratio: 1.99, 95.0% CI: 119–3,033; *P* = 0.01). Conversely, risk factors for VTE, including D-dimer, FDP, TAT III, F1 + 2, IL-6, IL-8, and granulocyte macrophage colony-stimulating factor levels, had no significant influence on survival.

## DISCUSSION

The novelty of this study was to assess the frequency of VTE detected by compression ultrasonography (CUS) and computed tomography (CT) angiography in patients with pancreatic carcinoma at the time of diagnosis. Asymptomatic VTE is difficult to detect without multimodal tests. Therefore, useful biomarkers are needed to detect asymptomatic VTE. Our study showed that 16.5% of chemo-naïve patients with advanced pancreatic carcinoma had VTE. However, the occurrence of VTE at the onset of pancreatic carcinoma did not appear to be closely related to symptoms of pancreatic carcinoma or patient prognosis. Patients with VTE frequently exhibited elevated D-dimer, FDP, and IL-6 levels. Moreover, the levels of TAT III and F1 + 2 were strongly associated with comorbid VTE. Hence, these factors may be useful for the early detection of VTE in patients with cancer.

VTE is the second leading cause of death in patients with pancreatic carcinoma and is associated with a shorter OS [9, 10]. Japanese patients with VTE are almost always asymptomatic and rarely have comorbid symptoms of VTE. A systematic review of patients with pancreatic carcinoma [11] reported that the incidence of VTE was 5.0–36.0%, representing a 50-fold increase in VTE rate in the population. The same study [11] reported that the rate of symptomatic VTE was 10.0%, whereas the rate in our study was 2.9%. In another study [12], the presence of VTE tended to be associated with age, sex, and Eastern Cooperative Oncology Group performance status. However, no specific clinical factors have been confirmed to be associated with VTE in patients with pancreatic carcinoma.

Elevated levels of certain markers (e.g., factor VII, IL-8, and plasminogen activator inhibitor-1) have been associated with an increase in thrombotic risk [13–15]. Moreover, initial reports [16, 17] also suggest that elevated levels of D-dimer, C-reactive protein, and F1 + 2 are risk factors for VTE in patients with cancer. In our study, elevated D-dimer, IL-8, and F1 + 2 levels were strongly associated with the occurrence of VTE. Furthermore, coagulant factors FDP and TAT III were associated with an increased risk of developing VTE in chemo-naïve patients with advanced pancreatic carcinoma. These factors may be especially important because the interrelation between cancer, coagulation activation, and secondary fibrinolysis is well known. Therefore, monitoring a combination of these factors may be a highly sensitive method for the early detection of VTE in patients with cancer.

Genetic variation is also a significant determinant of thrombotic risk [18–20]. Five inherited thrombophilias (Factor V Leiden, prothrombin gene mutation [G20210A], and protein C, protein S, and antithrombin deficiency) underlie a minority of VTE cases. Recently, several large genome-wide association studies [21, 22] have identified single nucleotide polymorphisms associated with VTE. Next-generation sequencing has also yielded promising findings in several VTE studies [23, 24]. However, there is no consensus on which patients should benefit from this screening without inherited thrombophilias [25].

VTE is associated with increased morbidity and mortality in patients with cancer [10, 11, 26]. However, the premature development of VTE in patients with pancreatic carcinoma was not associated with a poor prognosis in this study. There are several potential explanations, such as the use of edoxaban in all patients with VTE, asymptomatic VTE may not affect the prognosis of chemo-naïve patients with pancreatic carcinoma, and advances in chemotherapy may weaken the prognostic index of VTE. In a separate clinical trial, the prophylactic administration of low-molecular-weight heparin prevented the occurrence of VTE. However, it did not significantly influence prognosis, although elevated levels of VTE-associated factors (D-dimer and IL-6) were associated with a poor prognosis in previous studies [27–29]. In our study, VTE-associated factors, including D-dimer, were not associated with a poor prognosis, although carbohydrate antigen 19-9 (a well-known prognostic factor) was.

Our prospective study was limited by the fact that it was performed at a single institution with a sample size of 103 patients. Therefore, our results may not have sufficient statistical power. Other limitations include incomplete laboratory-based clinical thrombophilia testing, the absence of biochemical data to confirm structure function predictions, and limited family genetic studies.

In conclusion, our data indicated that approximately one-sixth of patients with advanced pancreatic carcinoma were complicated with VTE, which was asymptomatic in the majority of cases. Early detection of VTE may require diagnostic imaging techniques, such as CUS and CT angiography. However, factors such as D-dimer, TAT III, and F1 + 2 may be useful for the early detection of VTE in patients with pancreatic carcinoma. Additional prospective research is needed to validate these findings.

## MATERIALS AND METHODS

### Study approval

This prospective study was approved by the Institutional Review Board of the National Cancer Center (Tokyo, Japan). Informed consent has been obtained. Research was conducted in accordance with the Declaration of Helsinki. This study is registered in the University Hospital Medical Information Network clinical trials registry in Japan with the registration number UMIN000009474.

### Patient selection and blood sample collection

Chemotherapy-naive patients with histologically or cytologically confirmed advanced invasive ductal pancreatic carcinoma were prospectively enrolled in this study between January 2013 and October 2016. Patients administered anticoagulant or antiplatelet drugs were excluded. Details of the inclusion and exclusion criteria are provided in Supplementary Table 1. Prior to the initiation of cancer treatment, a detailed medical history was obtained from each participant. Additionally, a physical examination, recognition of symptoms of VTE (Supplementary Table 2), assessment of pretreatment baseline laboratory parameters, and a determination of baseline tumor status by CT of the chest, abdomen, and pelvis were performed (Supplementary Table 3). Baseline and posttreatment laboratory parameters were evaluated by performing peripheral blood sampling prior to and after the completion of treatment. The data collected included those pertaining to standard demographics; disease characteristics; and disease chronology, including the dates of initial treatment and death or final follow-up.

### Diagnosis of venous thromboembolism

CUS using the Doppler method [30] is the testing modality of choice in patients with pancreatic carcinoma, and has largely replaced venography for the diagnosis of proximal deep vein thrombosis. CT pulmonary angiography is the most widely used and evaluated test for diagnosing pulmonary embolism. It is currently the preferred diagnostic test because of its higher sensitivity and simpler reporting method. Eligible patients underwent CUS and dynamic CT, including pulmonary angiography, before commencing chemotherapy.

### Statistical analyses

The primary outcome was the incidence of concurrent VTE in patients with new-onset pancreatic carcinoma. The study had 90.0% statistical power to detect the incidence of VTE at a two-sided *P*-value of 0.05. It was calculated that the VTE rate was to be achieved in ≥15.0% of the 98 evaluable patients. The secondary outcomes were the associations between several tested biomarkers and the incidence of VTE and associated survival outcomes. Continuous variables were assessed for normal distribution using the Kolmogorov-Smirnov test. Results were expressed as the mean (± standard deviation) or median (range), as appropriate. The statistical significance of continuous variables was determined using the Student's *t*-test or Mann–Whitney *U* test, depending on the normality of the data. Median values were compared using the Kruskal-Wallis test. For categorical variables, the chi-square or Fisher's exact test was used, as appropriate. Survival curves were constructed using the Kaplan-Meier method. All statistical analyses were conducted using Statistical Package for the Social Sciences for Windows, software version 18.0 (SPSS Inc., Chicago, IL, USA). A *P* < 0.05 was considered statistically significant.

## SUPPLEMENTARY MATERIALS TABLES



## Abbreviations

CI: confidence interval
CT: computed tomography
CUS: compression ultrasonography
F1 + 2: prothrombin fragment 1 + 2
FDP: fibrin degradation product
IL: interleukin
OS: overall survival
TAT III: thrombin/antithrombin III complex
VTE: venous thromboembolism
