# Supplementary material for: Incidence and risk factors for venous thromboembolism in patients with pretreated advanced pancreatic carcinoma
Source: Oncotarget. 2018 Mar 30;9(24):16883–90. doi: 10.18632/oncotarget.24721 (PMC5908292; doi:10.18632/oncotarget.24721)
Supplement: Supplementary file 1 [file oncotarget-09-16883-s001.pdf]

Incidence and risk factors for venous thromboembolism in patients with pretreated advanced pancreatic carcinoma

SUPPLEMENTARY MATERIALS

Supplementary Table 1: Inclusion and exclusion criteria

**Inclusion criteria**
1. Aged ≥18 years<br>2. Histologically or cytologically confirmed advanced invasive ductal carcinoma<br>3. Previously untreated pancreatic carcinoma<br>4. ECOG PS of ≤2<br>5. Adequate hematological, hepatic, and renal function<br>6. Provision of written informed consent
**Exclusion criteria**
1. Thrombectomy, caval filter insertion, or use of a fibrinolytic agent to treat the current (index) episode of DVT and/or PE<br>2. Pretreatment with therapeutic doses of anticoagulant treatment (LMWH, unfractionated heparin, and fondaparinux per local labeling), oral direct anticoagulants, or vitamin K antagonists prior to enrollmentenroll in the study;<br>3. An Eastern Cooperative Oncology Group (ECOG) PS of 3 or 4 at the time of enrollment<br>4. Contraindication of dynamic CT using radiographic contrast agents<br>5. Active autoimmune disease or immunodeficiencies that require treatment with systemic immunosuppressive agents<br>6. Active infection that requires treatment with antibiotics or antiviral therapies<br>7. Any psychiatric condition that would prohibit the understanding or rendering of informed consent.

Abbreviations: CT, computed tomography; DVT, deep vein thrombosis; ECOG, Eastern Cooperative Oncology Group; LMWH, low-molecular-weight heparin; PE, pulmonary embolism; PS, performance status.

Supplementary Table 2: Symptom criteria

• Whole leg swollen
• Calf swelling (≥3.0 cm larger than the asymptomatic side)
• Pitting edema confined to the symptomatic leg
• Heart rate >100 beats per minute
• Hemoptysis
• Dyspnea

Supplementary Table 3: Diagnostic criteria

1. CT angiography
• Intraluminal filling defect on a staging CT scan of the inferior vena cava or iliac veins
• Non-compressible venous segment in the popliteal, iliac, common femoral, or femoral vein
• Intraluminal filling defect in segmental or more proximal branches on spiral CT pulmonary angiography
2. CUS using the Doppler method
• A combination of extended CUS of the proximal deep veins and CUS of the distal deep veins of the leg.
3. Diagnosis by independent clinicians

Abbreviations: CT, computed tomography; CUS, compression ultrasonography.
